# Characteristics of employees with lower health interest in a Japanese IT company: a cross-sectional study using Breslow’s health practice index

**DOI:** 10.1093/joccuh/uiaf057

**Published:** 2025-11-04

**Authors:** Yumiko Iwase, Rikuya Hosokawa

**Affiliations:** Department of Human Health Sciences, Graduate School of Medicine, Kyoto University, Kyoto, Japan; Graduate School of Nursing for Health Care Science, Kyoto Prefectural University of Medicine, Kyoto, Japan

**Keywords:** occupational health, health behavior, health knowledge, attitudes, practice, Breslow’s Health Practice Index

## Abstract

**Objectives**: This study examined the characteristics of employees with low health interest using Breslow’s Health Practice Index (HPI).

**Methods**: A cross-sectional study of 2260 employees of a Japanese IT company was conducted in 2023. The association between the Health Interest Scale (HIS; range 0-36) and HPI (range 0-7), a predictor of healthy longevity, was examined using multiple linear and logistic regression. Additional analyses were conducted using median-split HIS groups (low: 0-23; high: 24-36).

**Results**: HIS was significantly lower among men, younger and unmarried individuals, and those without an appropriate body weight, but positively associated with HPI (β = .254, *P* < .001). HPI was significantly higher among less sedentary workers (β = .07, *P* < .001), non–management staff (β = .04, *P* < .05), and married individuals (β = .06, *P* < .05). HIS was associated with 6 of 7 health behaviors except Not snacking. After adjustment for HIS, women had higher odds of Not smoking (OR = 5.52; 95% CI, 2.96-10.3; *P* < .001) and Moderate use of alcohol (OR = 2.03; 95% CI, 1.33-3.09; *P* < .05). Median-split analysis confirmed these results.

**Conclusions**: Interventions are needed to increase health interest among younger individuals, men, and those who are unmarried or without an appropriate body weight. Sedentary workers, managerial staff, and unmarried individuals showed lower adherence to HPI after adjustment for health interest, indicating the need for focused workplace interventions. Health interest was positively associated with HPI, but no significant association was found for snacking, which requires further investigation.

## Teaser

Key points:

- **What is already known on this topic**: National policy promotes health practices among individuals with low health interest. Population-based studies of adults have shown associations between health interest and basic demographic factors such as age and gender. However, the characteristics of populations with low health interest remain insufficiently clarified. In working populations, long working hours and occupational demands limit adherence to health practices. These findings underscore the need to examine such populations in occupational settings.
- **What this study adds**: Whereas previous studies have reported lower Health Interest Scale (HIS) scores among younger adults and men, this study additionally identified such lower scores in unmarried individuals and those outside the appropriate weight range. Even with adjustment for HIS, sedentary occupations and managerial positions were associated with lower adherence to Breslow’s Health Practice Index (HPI). Of the 7 health practices, only snacking showed no significant association with HIS. After controlling for HIS, women still had higher odds of not smoking and of practicing moderate alcohol use, underscoring sex-specific differences in health practices.
- **How this study might affect research, practice, or policy**: Interventions should prioritize younger employees, men, unmarried individuals, and those without an appropriate body weight, who showed lower health interest. Sedentary and managerial employees demonstrated lower adherence to health practices independent of health interest, highlighting the need for workplace-targeted strategies. Marked sex differences, particularly in smoking and alcohol use, underscore the importance of designing gender-sensitive interventions.

## 1. Introduction

According to the World Health Organization, noncommunicable diseases (NCDs) cover a wide range of chronic conditions, including cancer, diabetes, cardiovascular diseases, respiratory diseases, and mental health disorders.^1^ They cause about 41 million deaths annually, or 74% of all deaths. Roughly 17 million occur before age 70, with serious social consequences.^2^ In Japan, NCDs account for nearly 82% of deaths,^3^ showing the urgent need for prevention. These diseases are strongly associated with lifestyle factors such as unhealthy diet, physical inactivity, smoking, and excessive alcohol use.^2^ Encouraging healthy behaviors is essential for preventing disease and maintaining a longer, healthier life. To address these issues, the Japanese government launched the Health Japan 21 initiative to promote healthier lifestyles, reduce health disparities, and extend healthy life expectancy.^4^ However, a survey by the Ministry (2024) found that 1 in 4 Japanese citizens expressed “no intention to improve” their dietary or exercise behaviors.^5^ In particular, working-age individuals often cite “being too busy with work” as the main barrier to maintaining healthy dietary habits, with 48.5% of those in their 30s, 45.3% in their 40s, and 32.9% in their 50s reporting this reason. Additionally, recent findings from a mixed-methods study of Japanese employees indicate that even those with motivation to improve their health may struggle to sustain behavioral changes without individualized support and feedback.^6^ Although the national policy, Health Japan 21 (the third term), emphasizes the need for intervention targeting individuals with low health interest,^7^ the definition and identification of this population group remain insufficiently established.

To support such efforts, the Health Interest Scale (HIS) was developed to measure individuals’ concern with health.^8^ The HIS consists of 3 subscales: health consciousness, health motivation, and health value. Previous studies using the HIS have identified associations between health interest and basic demographic factors such as gender and age.^8^^,^^9^ Furthermore, some studies have reported no significant association between health interest and alcohol consumption,^10^ whereas others have indicated that individuals with low health interest may engage in problematic drinking regardless of their socioeconomic status.^9^ A recent study using the Interest in Health Scale (IHS), a rescored version of the original Health Interest Scale (HIS), identified low-health-interest groups, which were more common among younger men and individuals with lower income.^11^ These findings suggest that the relationship between health interest and health behavior may differ by behavioral domain, underscoring the need for further empirical investigation.

In this context, the Health Practice Index (HPI) provides a useful behavioral indicator. The HPI is based on the “seven health practices” proposed by Breslow et al in the 1970s.^12^ Prior research has demonstrated that a greater number of these practices is associated with lower mortality rates^12^^,^^13^ and better subjective health and functioning. Evidence from Japan further supports the validity of the HPI: higher scores have been linked to fewer subjective symptoms and greater work satisfaction,^14^ and a lower prevalence of metabolic risk factors among younger workers.^15^

Although previous studies have clarified the associations between health interest and basic demographic factors such as age and gender, the characteristics of individuals with low health interest remain insufficiently understood. Since working-age individuals often face structural barriers such as long working hours, sedentary tasks, and workplace hierarchies, examining low-health-interest individuals in this population is particularly relevant. These occupational factors may reduce the practice of health behaviors independent of health interest, underscoring the need to clarify the characteristics of employees with low health interest.

## 2. Methods

### 2.1. Study design and participants

This cross-sectional study was conducted among employees of a large information technology (IT) company in Japan. The survey targeted employees assigned to Tokyo and the surrounding metropolitan area who received annual health check-ups at a designated facility during fiscal year (FY) 2023, and 4000 paper-based questionnaires were distributed to ensure sufficient data collection based on the sample size estimation.

### 2.2. Sample size

Sample size was calculated using G*Power 3.1.9.7^16^ for a multiple regression model with 7 predictors. The effect size was set at f^2^ = 0.15, which corresponds to a medium effect size as defined by Cohen,^17^ assuming no prior data. With alpha = .05 and 1 − beta = .80, the minimum required sample size was 103 participants. Considering the gender ratio (female: 18.7%) in FY2022, 103 participants per gender were required for subgroup analyses, resulting in a total of 551 participants:


$$n= \frac{103}{0.187}\ \left(n:\mathrm{Sample}\ \mathrm{size}\right). $$

Additionally, based on a 2020 web-based survey conducted by the target company, the response rate was estimated to be approximately 60%. Adjusting for this response rate, the final required sample size was calculated as:


$$n= \frac{551}{0.60}\ \left(n:\mathrm{Sample}\ \mathrm{size}\right)=\textrm{approximately}\ 920. $$

Women constituted 18.7% of employees, and this distribution would have provided about 180 valid female responses, which was inadequate for stratified analyses by sex, age, and occupational characteristics. Stable subgroup analyses required 300-400 valid female responses, and the necessary distribution was therefore estimated at 3500-4000. Accordingly, 4000 paper-based questionnaires were distributed.

### 2.3. Outcome variable

In this study, the HPI was employed as the outcome variable. It consists of 7 health practices: not smoking, moderate alcohol use, adequate sleep, eating breakfast, regular exercise, maintaining proper weight, and not snacking. The reliability of the HPI was confirmed through a test–retest study, in which a majority of participants provided consistent responses across demographic, psychological, and physical health items.^18^^,^^19^ Its validity was supported by comparison between survey responses and clinical records, showing reasonable agreement in health status ratings.^19^

Component definitions of the HPI are summarized in Table S1.

### 2.4. Independent variable

HIS was treated as a continuous variable based on total scores, with each item rated on a 3-point scale, yielding a maximum of 36 points. The scale consists of 12 items. In the absence of established cutoff values for the HIS, the total score was treated as a continuous variable to maximize the information in the data. The 12 items are organized into 3 subscales: health consciousness, health motivation, and health value. Internal consistency was high, with Cronbach’s alpha reported as .85. Confirmatory factor analysis showed good model fit.^9^ The indices were as follows: Goodness-of-Fit Index (GFI) = 0.932, Adjusted Goodness-of-Fit Index (AGFI) = 0.896, Comparative Fit Index (CFI) = 0.936, and Root Mean Square Error of Approximation (RMSEA) = 0.079. Item content by subscale is provided in Table S2.

### 2.5. Covariates

Covariates included demographic attributes and background factors such as gender, age, job titles, job positions, living with family, and marital status. Job titles were collected across various roles and then categorized into 2 groups: “sedentary job categories” (system engineer [SE], customer engineer [CE], and office workers) and “non-sedentary job categories” (all others). This classification follows prior research indicating a higher risk of disease associated with sedentary occupations.^20^Management staff are those holding positions equivalent to Director or above Senior Advisor, corresponding to the International Standard Classification of Occupations 08 (ISCO-08) major group 1 (Managers).^21^

### 2.6. Statistical analyses

We conducted a comparative analysis of the HPI and HIS scores based on the participants’ characteristics. Independent-sample *t* tests were conducted using demographics such as gender, age, job category, job position, living situation, and marital status. Additionally, 1-way analysis of variance (ANOVA) examined significant differences in mean scores among 3 age groups: under 39 years, 40-49 years, and 50 years or older.

To assess the association between HIS and HPI scores, correlation coefficients were calculated, including correlations with age. Simple regression analyses were performed with HPI as the dependent variable, first using HIS as the independent variable, and then using demographic attributes. A multiple regression analysis was also conducted with HPI as the dependent variable, HIS as the independent variable, and demographics as covariates to further explore this association.

For each health behavior within the HPI, univariable logistic regression analyses were performed to assess the association with health interest. Multivariate logistic regression analyses were also conducted for each health behavior, using the HIS score and demographic attributes as independent variables, with relevant background factors as covariates. Since no established cutoff value exists for the HIS score and its distribution was approximately normal, we treated it as a continuous variable in the primary analyses. This approach allowed us to retain more statistical information and improve the precision of the estimated associations.

As a supplementary analysis, the total HIS score was additionally categorized in 2 ways: (1) using a median split (low: 0-23; high: 24-36), and (2) into quintiles. Logistic regression models were then conducted using these categorical HIS variables to examine the robustness of the associations and to explore potential nonlinear relationships between health interest and health behavior adherence. These analyses also aimed to identify subgroups with high interest but inconsistent health behavior practices. All statistical analyses were performed using SPSS version 28, with statistical significance set at *P* < .05.

## 3. Results

### 3.1. Characteristics of the participants

**Figure 1:**
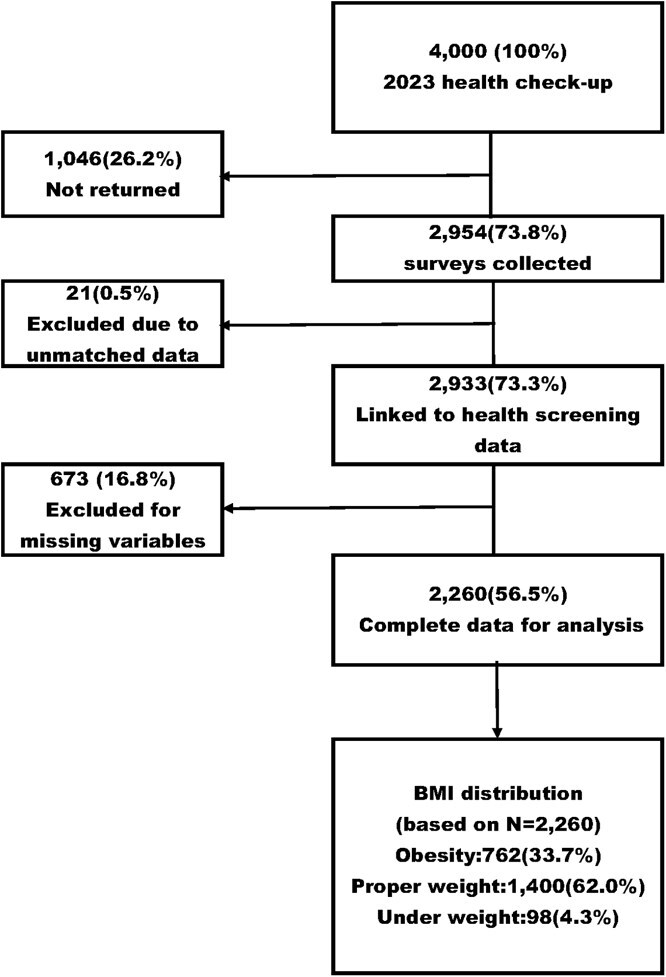
Flow diagram of the study participants. Percentages in the flow diagram are based on the total number of questionnaires distributed (*n* = 4000). Body mass index (BMI) category percentages are based on the final analytical sample (*n* = 2260). BMI categories (kg/m^2^) were defined as follows: Obesity: BMI ≥ 25; Proper-Weight: 18.5 ≤ BMI < 25; Under-Weight: BMI < 18.5.

During the annual health check-up period from November 27, 2023, to January 17, 2024, 4000 questionnaires were distributed, and 2954 responses were received (response rate: 73.9%). After excluding 673 cases with missing data, 2260 individuals (56.5%) were included in the final analysis (Figure 1).

The participants’ demographic characteristics are presented in Table 1: mean age was 50.7 years (SD = 6.8), and mean body mass index (BMI) was 24.0 kg/m^2^ (SD = 3.8). Most participants were male (85.3%), of whom 38.1% were employed as systems engineers. The majority held non–management staff positions (89.2%), and 56.6% were involved in sedentary work. Most participants resided with their family (73.3%), and 75.6% were married. Finally, 59.1% were aged 50 years or older, 35.8% were aged 40-49 years, and 5.1% were younger than 40 years.

**Table 1:** Participant demographics (*n* = 2260).

**Variable**	**Categorization criteria**	***n***	**%**
**Sex**	Male	1928	85.3
	Female	332	14.7
**Job title**	Sales staff	247	10.9
	Office worker	419	18.5
	R&D staff	861	38.1
	SE or CE	546	24.2
	Management staff^a^	187	8.3
**Job position**	General staff	1485	65.7
	Manager	301	13.3
	Senior manager	229	10.1
	Director	181	8.0
	Above senior advisor	64	2.8
**Management position**	Non–management staff	2015	89.2
	Management staff	245	10.8
**Job category**	Less sedentary staff	980	43.4
	More sedentary staff^b^	1280	56.6
**Living with family**	Yes	1657	73.3
	No	603	26.7
**Marital status**	Yes	1709	75.6
	No	551	24.4
**Age group, y**	Under 40	115	5.1
	40-49	809	35.8
	50+	1336	59.1
**BMI category, kg/m** ^**2**^	Underweight (BMI <18.5)	98	4.3
	Proper weight (BMI 18.5 to <25)	1400	62.0
	Obesity (BMI ≥25)	762	33.7
**Health practice**	Nonsmoker	1907	84.4
	Moderate exercise (≥60 min/d walking)	201	8.9
	Moderate use of alcohol (≥1 alcohol-free day/wk)	1895	83.8
	Adequate sleep (≥7 h/night)	645	28.5
	Not snacking	1877	83.1
	Eating breakfast	1469	65.0
	Proper weight	1400	61.9
**Variable**			
**Age, mean (SD), y**	50.7 (6.8)		
**BMI, mean (SD), kg/m** ^**2**^	24.0 (3.8)		
**HPI** ^**c**^			
**Mean (SD)**	4.16 (1.11)		
**Min/max (median)**	0/7 (4.00)		

Abbreviations: BMI, body mass index; CE, customer engineer; SE, system engineer.

a Management staff = Director or above Senior Advisor.

b More sedentary staff = SE, CE, or office worker.

c HPI, Health Practice Index (Breslow’s Health Practice Index), representing the total number of 7 health practices (range 0-7), with higher scores indicating healthier behaviors.

The BMI of 62.0% of participants was normal, 33.7% were obese, and 4.3% were underweight. The mean HPI score among all participants was 4.16 (SD = 1.11), with scores ranging from 0 to 7 and a median of 4.00. Regarding individual health practices, 84.4% were nonsmokers, 83.8% had at least 1 alcohol-free day per week, 65.0% ate breakfast regularly, and 61.9% maintained a proper weight. Additionally, 28.5% reported adequate sleep (≥7 hours per night) and 8.9% engaged in moderate daily exercise (≥60 minutes of walking), and 83.1% reported not snacking.

### 3.2. Comparison of the HIS scores according to the participants’ characteristics

Table 2 summarizes the associations between HIS scores and participant characteristics. The mean HIS score among all participants was 23.01 (SD = 5.03), with scores ranging from 3 to 36 and a median of 23.00. Higher HIS scores were observed among participants aged ≥50 years compared with those under 50 (*P* < .001), and among females compared with males (*P* < .001). Married individuals and those living with family had significantly higher scores than their unmarried or independently living counterparts (both *P* < .05).

**Table 2:** Health Interest Scale (HIS) scores by demographic and occupational characteristics

**Variable**	***n***	**%**	**Mean**	**SD**	**95% CI**		**Comparison with under-40s** ^**a**^		**Comparison with 40-49** ^**a**^	
					**Lower**	**Upper**	**Mean difference**	***P* value**	**Mean difference**	***P* value**
**Age group**										
**Under 40**	115	5.1	21.7	4.79	20.8	22.6	—	—	−0.75	.29
**40-49**	809	35.8	22.4	4.95	22.1	22.8	0.75	.29	—	—
**50+**	1336	59.1	23.5	5.05	23.2	23.7	1.78	**<.001**	1.03	**<.001**
**Total**	2260	100	23.0	5.03	22.8	23.2	—	—	—	—
**Variable**		***n***	**%**		**Mean**	**SD**	***t* value**		***P* value**	
**Sex**										
**Female**		332	14.70		24.13	5.00				
**Male**		1928	85.30		22.82	5.01	4.39		**<.001**	
**Job category**										
**Less sedentary staff**		980	43.40		23.20	5.11	1.60		.11	
**More sedentary staff**^**b**^		1280	56.60		22.86	4.97				
**Management position**										
**Non–management staff**		2015	89.20		22.99	5.07	−0.53		.60	
**Management staff**^**c**^		245	10.80		23.17	4.73				
**Married**										
**Yes**		1709	75.60		23.20	4.99	2.75		**<.05**	
**No**		551	24.40		22.50	5.15				
**Living with family**										
**Yes**		1657	75.30		23.21	4.94	3.05		**<.05**	
**No**		603	26.70		22.46	5.23				
**Proper weight (BMI 18.5 to <25 kg/m** ^**2**^ **)**										
**Yes**		860	40.70		23.65	4.92	−7.86		**<.001**	
**No**		1400	59.30		21.96	5.04				
**All participants**		2260	100.0		23.01	5.03	3/36 (min/max)		23.0 (median)	

Abbreviations: ANOVA, analysis of variance; BMI, body mass index; CE, customer engineer; HIS, Health Interest Scale; SE, system engineer.

a *t* test or 1-way ANOVA was used. Bold values indicate statistical significance (*P* < .05).

b More sedentary staff = SE, CE, or office worker.

c Management staff = Director or above Senior Advisor. The bottom row (“All participants”) shows overall HIS statistics (n = 2260: mean, SD, median, min–max).

Participants with normal body weight scored significantly higher than those classified as underweight or overweight (*P* < .001). Regarding health behaviors, nonsmokers had higher HIS scores than current smokers (*P* < .001), and those walking ≥60 minutes per day scored higher than those walking less (*P* < .05). Similarly, individuals who slept ≥7 hours per night or regularly ate breakfast (daily or occasionally) showed significantly higher scores compared with their comparison groups (both *P* < .05 and *P* < .001, respectively). By contrast, snacking frequency was not significantly associated with HIS scores (*P* = .895). Group differences in HPI across participant characteristics are summarized in Table S3.

### 3.3. Multiple regression analysis of the HPI scores based on the HIS scores and the participants’ characteristics

Multiple regression analysis was conducted to examine the associations between HIS and HPI scores, adjusting for demographic and occupational factors. As shown in Table 3, HIS scores were positively associated with HPI scores in the multiple regression model (β = .254, *P* < .001). Higher HPI scores were also associated with having a less sedentary occupation (β = .075, *P* < .001), having a spouse (β = .060, *P* < .05), and being non–management staff (β = .045, *P* < .05).The correlation matrix among HPI, HIS, and age is shown in Table S4.

**Table 3:** Multivariable regression analysis: coefficients for HPI by HIS scores and demographics (*n* = 2260).^a^

**Variable**		**Unstandardized coefficient**		**Standardized coefficient**	***t* value**	***P* value**	**95% CI for B**	
		**B**	**SE**	**β**			**Lower**	**Upper**
**Health interest score by HIS**	(Continuous variable)	0.06	0.00	.254	12.32	**<.001**	0.05	0.06
**Age, y**	(Continuous variable)	0.00	0.00	.01	0.66	.51	0.00	0.01
**Sex**	Female (ref: Male)	0.08	0.07	.03	1.25	.21	−0.05	0.21
**Job category**	Less sedentary staff (ref: More sedentary staff^b^)	0.17	0.05	.07	3.64	**<.001**	0.08	0.26
**Management position**	Non–management staff (ref: Management staff^c^)	0.16	0.07	.04	2.18	**<.05**	0.02	0.30
**Living with family**	Yes (ref: No)	0.02	0.06	.01	0.25	.80	−0.11	0.14
**Married**	Yes (ref: No)	0.15	0.07	.06	2.35	**<.05**	0.03	0.28

Abbreviations: CE, customer engineer; HIS, Health Interest Scale; HPI, (Breslow’s) Health Practice Index; SE, system engineer; VIF, variance inflation factor.

a Dependent variable: HPI (continuous). Independent variable: HIS score. Reference categories: male, more sedentary staff, management staff, not living with family, unmarried. Multiple linear regression was used (*R* = 0.284, *R*^2^ = 0.081). All VIF values were below 2.0 (range: 1.03-1.59), indicating no serious multicollinearity. Bold values indicate statistical significance (*P* < .05).

b More sedentary staff = SE, CE, or office worker.

c Management staff = Director or above Senior Advisor.

No significant associations were found for age, gender, or living with family. Additionally, when participants were divided into 2 groups based on the median HIS score (low: 0-23; high: 24-36), multiple regression analysis revealed that individuals in the high HIS group had significantly higher HPI scores (β = .210, *P* < .001). Similar to the main analysis, having a less sedentary occupation (β = .074, *P* < .001), being non–management staff (β = .046, *P* < .05), and having a spouse (β = .067, *P* < .05) were associated with higher HPI scores. No significant associations were found for gender, age, or living with family. Individuals in the high HIS group demonstrated HPI scores approximately 0.21 points higher than those in the low HIS group.

### 3.4. Univariable logistic regression analysis of lifestyle behaviors based on the HIS scores

Univariable logistic regression analysis showed that higher HIS scores were significantly associated with 6 of Breslow’s 7 health practices, with the strongest association observed for nonsmoking (OR = 1.12; 95% CI, 1.10-1.15; *P* < .001). No significant association was found for snacking (*P*= .90).Simple linear regression coefficients for HPI are listed in Table S5. Univariable logistic regression results for HIS by demographics are provided in Table S6.

### 3.5. Multivariable logistic regression analysis of lifestyle behaviors based on the HIS scores and the participants’ characteristics

As shown in Tables 4 and 5, higher HIS scores were significantly associated with 6 of the 7 health behaviors: moderate alcohol use (OR = 1.03; 95% CI, 1.01-1.05; *P* < .05), not smoking (OR = 1.12; 95% CI, 1.09-1.15; *P* < .001), adequate sleep (OR = 1.03; 95% CI, 1.01-1.04; *P* < .001), daily breakfast (OR = 1.07; 95% CI, 1.05-1.09; *P* < .001), moderate exercise (OR = 1.04; 95% CI, 1.01-1.07; *P* < .05), and proper weight (OR = 1.07; 95% CI, 1.05-1.09; *P* < .001). No association was found with low snacking frequency (*P* = .40).

**Table 4:** Multivariable logistic regression analysis: odds ratios for HIS by demographics (*n* = 2260).^a^

**Variable**	**Moderate use of alcohol**				**Not smoking**				**Adequate sleep**				**Eating breakfast**			
	**OR**	**95%CI**		***P* value**	**OR**	**95% CI**		***P* value**	**OR**	**95% CI**		***P* value**	**OR**	**95% CI**		***P* value**
		**Lower**	**Upper**			**Lower**	**Upper**			**Lower**	**Upper**			**Lower**	**Upper**	
**HIS (continuous)**	1.03	1.01	1.05	**<.05**	1.12	1.09	1.15	**<.001**	1.03	1.01	1.04	**<.001**	1.07	1.05	1.09	**<.001**
**Sex**																
**Male**	Ref.				Ref.				Ref.				Ref.			
**Female**	2.03	1.33	3.09	**<.05**	5.52	2.96	10.3	**<.001**	1.28	0.99	1.67	.06	1.03	0.80	1.34	.81
**Age, y (continuous)**	0.97	0.95	0.98	**<.001**	0.99	0.97	1.00	.14	0.97	0.96	0.98	**<.001**	1.05	1.03	1.06	**<.001**
**Job category**																
**More sedentary staff**^**b**^	Ref.				Ref.				Ref.				Ref.			
**Less sedentary staff**	1.15	0.91	1.45	.25	1.28	1.01	1.63	**<.05**	1.39	1.15	1.67	**<.05**	1.17	0.98	1.41	.09
**Management position**																
**Management staff**^**c**^	Ref.				Ref.				Ref.				Ref.			
**Non–management staff**	1.66	1.21	2.30	**<.05**	1.64	1.17	2.31	**<.05**	1.14	0.84	1.54	.41	1.28	0.96	1.71	.09
**Living with family**																
**No**	Ref.				Ref.				Ref.				Ref.			
**Yes**	0.87	0.62	1.21	.41	1.05	0.73	1.50	.80	0.96	0.74	1.26	.79	1.22	0.95	1.57	.11
**Married**																
**No**	Ref.				Ref.				Ref.				Ref.			
**Yes**	0.78	0.55	1.12	.15	1.05	0.73	1.50	.80	1.42	1.08	1.88	**<.05**	1.59	1.23	2.05	**<.001**

Abbreviations: CE, customer engineer; HIS, Health Interest Scale; SE, system engineer; OR, odds ratio; CI, confidence interval.

a Dependent variables: each health behavior (binary). Independent variable: HIS score (continuous). Reference categories: male, more sedentary staff, management staff, not living with family, unmarried. Multivariable logistic regression was used. Bold values indicate statistical significance (*P* < .05).

b More sedentary staff = SE, CE, or office worker.

c Management staff = Director or above Senior Advisor.

Several demographic and occupational variables remained independently associated with health behaviors after controlling for HIS. Female participants were more likely to be nonsmokers (OR = 5.52, 95% CI, 2.96-10.27; *P* < .001) but less likely to report low snacking (OR = 0.36; 95% CI, 0.27-0.47; *P* < .001) or moderate exercise (OR = 0.46; 95% CI, 0.26-0.78; *P* < .05). Older age was positively associated with daily breakfast consumption (OR = 1.05; 95% CI, 1.03-1.06; *P* < .001), moderate exercise (OR = 1.06; 95% CI, 1.04-1.09; *P* < .001), and low snacking (OR = 1.02; 95% CI, 1.00-1.03; *P* < .05); however, it was negatively associated with adequate sleep (OR = 0.97; 95% CI, 0.96-0.98; *P* < .001). Married participants were more likely to eat breakfast (OR = 1.59; 95% CI, 1.23-2.05; *P* < .001). Those involved in less sedentary occupations exhibited higher odds of being nonsmokers (OR = 1.28; 95% CI, 1.01-1.63; *P* < .05), getting adequate sleep (OR = 1.39; 95% CI, 1.15-1.67; *P* < .05), and maintaining proper weight (OR = 1.21; 95% CI, 1.02-1.45; *P* < .05). General staff were more likely to report moderate alcohol use (OR = 1.66; 95% CI, 1.21-2.30; *P* < .05) compared with managers but less likely to report low snacking (OR = 0.55; 95% CI, 0.35-0.85; *P* < .05).

In the additional analysis using a median split of the total HIS score (low: 0-23; high: 24-36), individuals in the high HIS group were significantly more likely to engage in multiple health behaviors, including moderate alcohol use (OR = 1.41; *P* = .004), not smoking (OR = 2.74; *P* < .001), getting adequate sleep (OR = 1.38; *P* = .001), eating breakfast (OR = 1.58; *P* < .001), exercising moderately (OR = 1.39; *P* = .031), and maintaining a healthy weight (OR = 1.64; *P* < .001), compared with the low HIS group. These patterns remained consistent in the quintile analysis, indicating robustness of the associations. Multivariable logistic regression results by the median-split HIS groups are reported in Table S7 and Table S8. Multivariable linear regression coefficients for HPI by the median-split HIS groups are shown in Table S9.

In the multivariable logistic regression using HIS quintiles, participants in the highest quintile (28-36 points) had significantly higher odds of not smoking (OR = 3.91; 95% CI, 2.60-5.86; *P* < .001) than those in the lowest quintile (0-18 points). In contrast, no significant associations were observed for moderate use of alcohol (OR = 1.34; 95% CI, 0.94-1.91; *P* = .11) or not snacking (OR = 1.05; 95% CI, 0.74-1.49; *P* = .78), indicating that even among individuals with high health interest, some health behaviors were not consistently practiced. Multivariable logistic regression results by HIS quintiles are presented in Table S10 and Table S11.

## 4. Discussion

### 4.1. Characteristics of employees with low health interest

This study aimed to clarify characteristics of individuals exhibiting low health interest to support targeted workplace interventions. HIS scores were significantly lower among male and younger employees. These findings align with prior studies, indicating that these groups tend to exhibit lower health interest.^8^

Women tend to engage more frequently in health-protective behaviors,^8^ highlighting the importance of considering gender in intervention design. Among younger employees, internet-based health education^22^ has proven to be effective, indicating that tailored programs may therefore be effective for individuals with low health interest scores, particularly younger employees.

HIS scores were also higher among married individuals and those living with family. Supportive home environments promote health behaviors,^23^ and spouses often share health-related habits.^24^ These findings suggest that family-inclusive strategies may effectively increase health interest among working-age adults.^25^

### 4.2. Associations between sociodemographic and occupational factors and health behaviors

Several sociodemographic and occupational factors were significantly associated with health behaviors after adjusting for health interest. Job category, job position, and marital status remained significant, suggesting that workplace and personal characteristics influence health behaviors beyond health interest. Both marital status and family structure were associated with higher HPI scores in the present study. Married individuals exhibited different behavioral patterns compared with their unmarried counterparts. Prior studies have reported mixed findings: Lee et al^26^ found higher obesity rates among unmarried individuals, whereas Sato^27^ noted greater obesity in married women. Tanaka et al^28^ found that strong social networks were related to higher HPI scores and better subjective health, indicating the role of social support in reinforcing health behavior and mitigating family-related disadvantages.

Employees engaged in less sedentary work reported healthier behaviors compared with those in sedentary ones, even after controlling for health interest. Mizuno et al^29^ found that sedentary professionals, such as engineers, exhibited a higher risk of obesity. Conversely, Nam et al^30^ found no consistent link among professional work and cardiometabolic disease risk. These findings indicate that differences in health behaviors across job types remain to be examined in future research. Although further research is needed to clarify these differences, interventions targeting workplace physical conditions, including scheduled activity breaks^31^ and changes reducing sitting time, could help address job-related behavioral barriers.

### 4.3. Association between health interest and health behaviors

Individuals with higher health interest were more likely to engage in all health behaviors except snacking. For example, higher HIS scores were associated with being a nonsmoker, and women were more likely to refrain from smoking. These results align with prior findings identifying health consciousness as a contributor to smoking cessation.^32^ They support the need to combine health interest interventions with gender-specific approaches.

Higher HIS scores were related to moderate use of alcohol. Women were more likely to drink moderately, regardless of health interest. Previous studies have shown increased metabolic risk among male drinkers^33^ and greater problematic drinking among individuals exhibiting low health interest.^9^ Our findings suggest the need for targeted interventions addressing motivation and behavior, while paying attention to gender-specific patterns.

**Table 5:** Multivariable logistic regression analysis: odds ratios for HIS by demographics (*n* = 2260).^a^

**Variable**	**Not snacking**				**Moderate exercise**				**Proper weight**			
	**OR**	**95%CI**		***P* value**	**OR**	**95% CI**		***P* value**	**OR**	**95% CI**		***P* value**
		**Lower**	**Upper**			**Lower**	**Upper**			**Lower**	**Upper**	
**HIS (continuous)**	1.01	0.99	1.03	.40	1.04	1.01	1.07	**<.05**	1.07	1.05	1.09	**<.001**
**Sex**												
**Male**	Ref.				Ref.				Ref.			
**Female**	0.36	0.27	0.47	**<.001**	0.46	0.26	0.78	**<.05**	1.28	0.98	1.65	.07
**Age, y (continuous)**	1.02	1.00	1.03	**<.05**	1.06	1.04	1.09	**<.001**	0.99	0.97	1.00	.06
**Job category**												
**More sedentary staff**^**b**^	Ref.				Ref.				Ref.			
**Less sedentary staff**	0.86	0.68	1.08	.19	0.95	0.71	1.28	.74	1.21	1.02	1.45	**<.05**
**Management position**												
**Management staff**^**c**^	Ref.				Ref.				Ref.			
**Non–management staff**	0.55	0.35	0.85	**<.05**	1.38	0.83	2.30	.22	0.89	0.67	1.19	.43
**Living with family**												
**No**	Ref.				Ref.				Ref.			
**Yes**	0.69	0.49	0.96	**<.05**	1.20	0.80	1.81	.38	1.12	0.88	1.42	.37
**Married**												
**No**	Ref.				Ref.				Ref.			
**Yes**	0.92	0.66	1.28	.63	0.73	0.48	1.10	.13	1.21	0.94	1.55	.14

Abbreviations: CE, customer engineer; HIS, Health Interest Scale; SE, system engineer; OR, odds ratio; CI, confidence interval.

a Dependent variables: each health behavior (binary). Independent variable: HIS score (continuous). Reference categories: male, more sedentary staff, management staff, not living with family, unmarried. Multivariable logistic regression was used. Bold values indicate statistical significance (*P* < .05).

b More sedentary staff = SE, CE, or office worker.

c Management staff = Director or above Senior Advisor.

Regarding physical activity, higher HIS scores were associated with more active behavior. However, sedentary work constrained activity regardless of health interest. Health motivation, a subcomponent of HIS, has been linked to greater awareness of activity barriers.^34^ These findings highlight the importance of workplace strategies such as scheduled activity breaks.^31^

No significant association was found between health interest and snacking. As Hartmann et al^35^ noted, frequent snackers include health-conscious and relatively less health-conscious individuals, implying that snacking may not consistently reflect health interest.

All individual health behaviors except snacking were significantly more prevalent among those with higher health interest. In contrast, no significant difference was observed for snacking behavior. Individuals with higher health interest may deliberately choose healthy snacks such as nuts or fruit.

### 4.4. Implications for identifying low-health-interest groups and designing workplace health interventions

After adjusting for health interest, employees in sedentary jobs, management positions, or those who were unmarried exhibited significantly lower health behavior scores. These findings suggest that workplace health promotion should address both motivational factors and contextual barriers related to occupation and social conditions.

To support employees with low health interest, we need to understand the theoretical framework of health interest. The HIS is grounded in the theoretical framework of preventive health orientation proposed by Jayanti and Burns,^36^ which posits that psychological factors, including health value, consciousness, and motivation, precede behavioral action. Individuals with low HIS scores may not recognize the importance of health and fail to perceive preventive behaviors as relevant. For such individuals, educational approaches enhancing risk awareness and readiness for change can be effective. For instance, interventions based on the Health Belief Model^37^ can help increase perceptions of susceptibility, severity, benefits, and self-efficacy. However, such approaches are most effective when combined with other motivational and practical strategies.

Meanwhile, this study identified individuals with high health interest who nevertheless showed low engagement in healthy behaviors. Moorman and Matulich^38^ demonstrated that high motivation alone is insufficient to drive behavior change when abilities or opportunities are limited. A recent Japanese panel study also found that long working hours were associated with reduced physical activity, poorer diet, and shorter sleep duration.^39^ Unfavorable living environments and limited social support have also been linked to discontinuation of healthy behaviors.^40^ Future research should consider identifying and developing interventions for this group.

## 5. Strengths and limitations

This cross-sectional design precludes causal interpretation. In addition, although major occupational characteristics such as sedentary work and management status were analyzed, more detailed job-type differences could not be examined and should be addressed in future research. However, the study’s large sample and use of validated measures allowed for detailed analysis of factors associated with health interest among company employees.

## 6. Conclusion

The HIS was lower among younger individuals, men, unmarried participants, and those without an appropriate body weight. A higher HIS score correlated with a higher Breslow’s HPI. A higher HPI was observed among employees with less sedentary jobs, non–management staff, and married status. Six of the 7 health behaviors, excluding Not snacking, were associated with HIS. Even after adjustment for HIS, women were more likely than men to be nonsmokers and to practice moderate use of alcohol. Workplace health promotion should prioritize employees in sedentary jobs, in management roles, or those who are unmarried, with particular attention to sex-specific differences in health behaviors. The relationship between snacking and health interest requires further investigation.

## Supplementary Material

Web_Material_uiaf057

## Data Availability

The data supporting this study can be requested from the corresponding author. However, access is limited to protect the confidentiality of the participants.
